# Intravenous infusion of angiotensin II for treatment of cardiopulmonary bypass-induced vasoplegic shock after implantation of left ventricular assist device: a case report

**DOI:** 10.3325/cmj.2023.64.201

**Published:** 2023-06

**Authors:** Andrej Šribar, Verica Mikecin, Ivana Presečki, Davor Barić, Domagoj Marijančević, Jasminka Peršec

**Affiliations:** 1 Clinical Department of Anesthesiology, Reanimatology and Intensive Care Medicine Dubrava University Hospital, Zagreb, Croatia; 2 Zagreb University School of Dental Medicine, Zagreb, Croatia; 3 Department of Cardiac Surgery, Dubrava University Hospital, Zagreb, Croatia; 4 Clinical Department of Laboratory Diagnostics, Sestre Milosrdnice University Hospital Center, Zagreb, Croatia

## Abstract

We report on the first successful treatment of severe pharmacoresistant vasoplegic syndrome with angiotensin II acetate (ATII) in Croatia. ATII is a novel drug used to treat severe vasoplegic shock resistant to the administration of catecholamines or alternative vasopressors such as vasopressin or methylene blue. A 44-year-old patient with secondary toxic cardiomyopathy developed severe cardiopulmonary bypass-induced vasoplegic shock after scheduled implantation of a left-ventricular assist device. The cardiac output was maintained, but systemic vascular resistance (SVR) was extremely low. The patient had an inadequate reaction to the administration of high doses of norepinephrine (up to 0.7 µg/kg/min) and vasopressin (0.03 IU/min). At postoperative intensive care unit (ICU) admission, serum renin levels were unmeasurably high (>330 ng/L), and infusion of ATII 20 ng/kg/min was initiated. Soon after the start of the infusion, blood pressure increased. Vasopressin infusion was stopped, while the norepinephrine dose was decreased from 0.7 to 0.15 µg/kg/min. Serum lactate, mixed venous saturation, and glomerular filtration rate markedly improved. The patient was extubated 16 h after the ICU admission. Twenty-four hours after the start of the ATII infusion, serum renin dropped to 255 ng/L, and laboratory findings further improved. On postoperative day 3, the norepinephrine infusion was stopped. On day 6, renin further dropped to 136 ng/L, and the patient was hemodynamically stable and discharged from the ICU. In conclusion, ATII favorably affected the patient’s vascular tone, enabling rapid hemodynamic stabilization and shortening the ICU and hospital stay.

Vasoplegic syndrome is characterized by normal or increased cardiac output and reduced systemic vascular resistance (SVR), which warrants the administration of vasopressors to achieve adequate organ perfusion pressure (1). In up to 50% of patients, vasoplegia is caused by the use of cardiopulmonary bypass (CPB) and is marked by pathophysiologic mechanisms similar to those of sepsis (2). The most common drugs used for vasoplegia treatment are alpha-adrenergic catecholamines and vasopressin. When these options fail, clinicians may use angiotensin II acetate (ATII), a potent novel vasopressor. Pharmacoresistant vasoplegic syndrome often arises in the cardiac surgery setting, where the use of CPB, together with systemic inflammatory response, causes malfunctioning angiotensin converting enzyme (ACE). A reduced endogenous production of angiotensin II in vasoplegic patients may be explained by the preoperative use of ACE inhibitors and a significant reduction in pulmonary blood flow during CPB (3). In patients with mechanical circulatory support, ventricular-arterial coupling becomes dysregulated due to a loss of pulsatility, and sympathetic vascular tone usually increases immediately after surgery. If increase in vascular tone is inadequate, pressor support will be needed in the perioperative period do maintain MAP and organ perfusion. When cathecholamines and vasopressin fail, ATII may be considered as a therapeutic option.

## Case report

We report on a case of a 44-year-old man with secondary toxic cardiomyopathy due to a history of alcohol and cocaine abuse. He was scheduled for the implantation of a left ventricular assist device (LVAD). Other comorbidities were multi-organ failure, hypothyroidism, pulmonary emphysema, and the presence of a cardiac resynchronization therapy defibrillator.

One day before the surgery, the patient was transferred to the cardiac ICU, where a pulmonary artery catheter was inserted, and continuous infusion of levosimendan (0.05-0.1 µg/kg/min) was initiated as per the institutional LVAD pre-surgery preparation protocol.

In the operating theater, after induction of general anesthesia and preoperative transesophageal echocardiography (TEE), implantation of LVAD (Heartmate III, Abbott Cardiovascular, Abbott Park, IL, USA) and annuloplasty of the tricuspid valve were performed. The cumulative CPB duration was 130 minutes. After weaning off CPB, the patient was extremely hypotensive, although TEE showed adequate right heart contractility (with continuous milrinone infusion of 0.6 µg/kg/min) and left-ventricular end-diastolic volume, correct cannula position, and adequate outlet cannula flow. According to the LVAD control console, cardiac output (CO) of 3 L/min was generated. It was determined that the culprit was loss of SVR, and infusion of norepinephrine was initiated (up to 1 µg/kg/min). Since it was impossible to achieve a mean arterial pressure (MAP)>50 mm Hg with infusion of norepinephrine alone, infusion of vasopressin was started at 0.03 IU/min with a gradual increase in MAP to 70 mm Hg. Norepinephrine infusion was gradually reduced to 0.7 µg/kg/min.

After hemodynamic stabilization, surgical hemostasis, and closure, the sedated and mechanically ventilated patient was transferred back to the cardiac ICU for further treatment and stabilization. At admission, he was hemodynamically unstable, with LVAD generating CO 3.1 L/min. Infusions of milrinone 0.6 µg/kg/min, norepinephrine 0.7 µg/kg/min, and vasopressin 0.03 IU/min were needed to achieve a MAP of 70 mm Hg. Initial lab results showed slightly reduced mixed venous saturation (SvO_2_, 49.5%) and elevated serum lactate levels (4.8 mmol/L). Oliguria was also present, requiring the administration of furosemide to maintain diuresis and decreased glomerular filtration rate (GFR, 29 mL/min/1.73m^2^).

Since extremely high doses of norepinephrine and vasopressin were needed to achieve adequate perfusion pressures, serum renin levels were measured. The first value was unmeasurably high –>330 ng/L (automated chemiluminescence immunoassay, IDS-iSYS analyzer, Immunodiagnostic System Holdings, Boldon, UK). A decision was made to initiate ATII infusion at a 20 ng/kg/min rate (Giapreza, La Jolla Pharmaceutical, San Diego, CA, USA).

Within the first three hours after the start of the ATII infusion, MAP increased to 100 mm Hg. Vasopressin infusion was decreased to 0.01 IU/min and stopped after four hours. Norepinephrine dose was reduced from 0.7 µg/kg/min to 0.3 µg/kg/min, and then further to 0.15 µg/kg/min over the night. MAP was maintained between 70 and 90 mm Hg. On postoperative day (POD) 1, the patient was extubated. His serum lactate levels (2.2 mmol/L) normalized, serum renin was reduced to 255 ng/L after 24 h of ATII infusion, SvO_2_ gradually increased (52.4%), and CO was preserved after a reduction in milrinone dose. ATII infusion was stopped in the evening of POD 1, 28 h after its start.

On POD 3, milrinone and norepinephrine infusions were stopped. CI was 2.2 L/min/m^2^, SvO_2_ (66%) and serum lactate levels (0.7 mmol/L) normalized, and GFR was improved (38 mL/min/1.73m^2^).

On POD 6, serum renin further dropped to 136 ng/L, with complete normalization of GFR and marked improvement in serum transaminases and gamma glutamyl transferase. The patient was hemodynamically stable and discharged from the ICU. The timeline of relevant events is shown in Table 1.

**Table 1:** The timeline of events*

22 days before surgery (November 2022)	• Patient admitted to department of cardiology due to heart failure presenting as dyspnea • Left ventricular ejection fraction 10%, diastolic dysfunction grade 4, severe mitral regurgitation, moderate pulmonary regurgitation due to pulmonary hypertension, and moderate to severe tricuspid regurgitation with complete atrioventricular block
1 day before surgery/admitted to the cardiac ICU	• Insertion of pulmonary artery catheter, central venous catheter, arterial cannula • Levosimendan infusion initiated
Day of surgery	• Severe vasoplegia after LVAD implantation and weaning off CPB; milrinone, norepinephrine, and vasopressin initiated in the operating room • Serum renin unmeasurably high • ATII started after ICU admission, vasopressin stopped after 4 hours, NE dose reduced
POD 1	• Patient extubated, normalization of lactate levels, increase in SvO_2_, ATII infusion stopped in the evening, further reduction of NE dose • Serum renin started dropping
POD 3	• Norepninephrine and milrinone stopped • Gradual improvement in SvO_2_ and GFR
POD 6	• Further reduction of serum renin levels and improvement in GFR, liver function tests, and SvO_2_ • Patient discharged from ICU and transferred to cardiac surgery department
POD 10	• Patient transferred from cardiac surgery ward to cardiology department
POD 46	• Patient discharged from the hospital

*Abbreviations: LVAD – left ventricular assist device; CPB –cardiopulmonary bypass; ATII – angiotensin II acetate; ICU – intensive care unit; NE – norepinephrine; SvO_2_ –venous oxygen saturation; GFR – glomerular filtration rate.

## Discussion

CPB-induced vasoplegia is characterized by inducible nitric oxide synthase stimulation and increased release of nitric oxide caused by inflammatory response to surgical stress, synthetic materials in the circuit, hemolyzed blood cells, and postischemic reperfusion injury. Also, phosphorylation of adrenergic receptors impairs catecholamine binding, thus blunting the response to catecholamines. These factors, together with acidosis, depletion of endogenous arginine-vasopressin, and cell membrane hyperpolarization secondary to K_ATP_ channel stimulation, lead to severe vasoplegia refractory to high doses of α_1_ agonists. Since the administered catecholamine dose is correlated with in-hospital mortality (4), several alternative options are available as adjuvant therapy to decrease norepinephrine requirements – vasopressin (5), methylene blue (6), and angiotensin II (7). Angiotensin II is an active form of peptide hormone angiotensin I. ATII was approved by the US Food and Drug Administration in 2017 and by the European Medicines Agency in 2019. It has a positive inotropic and chronotropic effect, and increases aldosterone and vasopressin levels, as well as norepinephrine release and catecholamine sensitivity. Compared with norepinephrine alone, the use of ATII to treat catecholamine-resistant vasoplegic shock in combination with norepinephrine is associated with a higher probability of achieving a MAP of 75 mm Hg after three hours and a greater reduction of the cardiovascular subcomponent of the SOFA score (8).

Our decision to administer ATII was guided by elevated serum renin (9), and its kinetics showed the patient’s favorable survival odds following ATII administration (10). There is still inconclusive evidence that ATII is superior to other vasopressors regarding hemodynamic response or decreased mortality (7,11), especially in the cardiac surgery setting, where there are only case reports or circumstantial evidence (3). However, this drug is considered safe (8) and can be used as a salvage vasopressor in vasoplegia.

## References

[1] Busse LW Barker N Petersen C Vasoplegic syndrome following cardiothoracic surgery—review of pathophysiology and update of treatment options. Crit Care 2020 24 36 10.1186/s13054-020-2743-8 32019600 PMC7001322

[2] Omar S Zedan A Nugent K Cardiac vasoplegia syndrome: pathophysiology, risk factors and treatment. Am J Med Sci 2015 349 80 8 10.1097/MAJ.0000000000000341 25247756

[3] Papazisi O Palmen M Danser AHJ The use of angiotensin ii for the treatment of post-cardiopulmonary bypass vasoplegia. Cardiovasc Drugs Ther 2022 36 739 48 10.1007/s10557-020-07098-3 33085026 PMC9270278

[4] Sacha GL Lam SW Wang L Duggal A Reddy AJ Bauer SR Association of catecholamine dose, lactate, and shock duration at vasopressin initiation with mortality in patients with septic shock*. Crit Care Med 2022 50 614 23 10.1097/CCM.0000000000005317 34582425

[5] Russell JA Walley KR Singer J Gordon AC Hébert PCCooper DJ, et alVasopressin versus norepinephrine infusion in patients with septic shock. N Engl J Med 2008 358 877 87 10.1056/NEJMoa067373 18305265

[6] Abdelazim R Salah D Labib HA El Midany AA Methylene blue compared to norepinephrine in the management of vasoplegic syndrome in pediatric patients after cardiopulmonary bypass: a randomized controlled study. Egypt J Anaesth 2016 32 269 75 10.1016/j.egja.2016.05.001

[7] Wieruszewski PM Wittwer ED Kashani KB Brown DR Butler SO Clark AM Angiotensin II infusion for shock: a multicenter study of postmarketing use. Chest 2021 159 596 605 10.1016/j.chest.2020.08.2074 32882250 PMC7856533

[8] Angiotensin II for the treatment of vasodilatory shock: enough data to consider angiotensin II safe? Critical Care. Available from: https://ccforum.biomedcentral.com/articles/10.1186/s13054-018-2006-0. Accessed: December 3, 2022. 10.1186/s13054-018-2006-0 PMC5902841 29661216

[9] Bellomo R Forni LG Busse LW McCurdy MT Ham KR Boldt DW Renin and survival in patients given angiotensin ii for catecholamine-resistant vasodilatory shock. a clinical trial. Am J Respir Crit Care Med 2020 202 1253 61 10.1164/rccm.201911-2172OC 32609011 PMC7605187

[10] Jeyaraju M McCurdy MT Levine AR Devarajan P Mazzeffi MA Mullins KE Renin kinetics are superior to lactate kinetics for predicting in-hospital mortality in hypotensive critically ill patients. Crit Care Med 2022 50 50 60 10.1097/CCM.0000000000005143 34166293

[11] Quan M Cho N Bushell T Mak J Nguyen N Litwak J Effectiveness of angiotensin ii for catecholamine refractory septic or distributive shock on mortality: a propensity score weighted analysis of real-world experience in the medical ICU. Crit Care Explor 2022 4 e0623 10.1097/CCE.0000000000000623 35072084 PMC8769135

